# Oleoylethanolamide Supplementation Reduces Inflammation and Oxidative Stress in Obese People: A Clinical Trial

**DOI:** 10.15171/apb.2018.056

**Published:** 2018-08-29

**Authors:** Laleh Payahoo, Yaser Khajebishak, Mohammad Asghari Jafarabadi, Alireza Ostadrahimi

**Affiliations:** ^1^Talented Student Center, Student Research Committee, Nutrition Research Center, Faculty of Nutrition and Food Science, Tabriz University of Medical Sciences, Tabriz, Iran.; ^2^Student Research Committee, Faculty of Nutrition and Food Science, Tabriz University of Medical Sciences, Tabriz, Iran.; ^3^Road Traffic Injury Research Center, Department of Statistics and Epidemiology, Faculty of Health, Tabriz University of Medical Sciences, Tabriz, Iran.; ^4^Nutrition Research Center, Faculty of Nutrition and Food Science, Tabriz University of Medical Sciences, Tabriz, Iran.

**Keywords:** Inflammation, Endocannabinoids, Obesity, Oleoylethanolamide, Oxidative stress

## Abstract

***Purpose:*** Obesity as a serious public health problem worldwide, results in the incidence of many chronic diseases. Obesity has been recognized as a chronic low-grade inflammation disorder. Altered endocannabinoid system tone is also involved in the pathogenesis of obesity. The present study aimed to investigate the effects of oleoylethanolamide supplementation on inflammatory biomarkers and oxidative stress in obese people.

***Methods:*** This randomized, double-blind, placebo-controlled clinical trial was carried out on 60 healthy obese people in 2016 in Tabriz, Iran. Eligible subjects were randomly divided into intervention (received daily, two 125 mg OEA capsules) and control groups (the same amounts of starch) and treated for 8 weeks. Blood samples (5 ml) were taken in fasting state at the baseline and at the end of the study. The concentrations of MDA and TAS were measured using a spectrophotometer. A high sensitive-C reactive protein level was measured by Immunoturbidimetry assay using the commercial kit. IL-6 and TNF-α levels were assayed by the ELISA method. The differences between groups were assessed by ANCOVA and statistical significance was determined at p<0.05.

***Results:*** Analysis was done on 56 participants who continued intervention until the end of the study. A significant decrease in the IL-6 and TNF-α serum concentrations was observed in the intervention group (p<0.001). Changes in other variables were undetectable (p>0.05).

***Conclusion:*** The use of OEA as a complementary pharmacotherapy agent could be effective in improving inflammation and oxidative stress in obese people. Future studies are needed to confirm the obtained results.

## Introduction

Obesity as a serious public health problem, results from an imbalance between energy intake and energy expenditure.^1^ Growing evidences have shown that there is an increase in the prevalence of obesity in the world.^2^ Iran is no exception in this regard. According to the second national integrated micronutrient survey in 2011 to 2015, about 40.3 and 19.2% of the Iranian population were overweight and obese, respectively.^3^ Obesity was recognized as a chronic low-grade inflammation.^4^ Chronic inflammation participates in the appearance of many adverse disorders such as diabetes, metabolic syndrome, some kinds of cancer and nonalcoholic fatty liver disease.^5-8^ It has been shown that the levels of pro-inflammatory cytokines and acute phase proteins such as C-reactive protein (CRP), interleukin-6 (IL-6) and tumor necrosis factor–alpha (TNF-α) increase in obese people.^9-11^

Besides inflammation, an altered endocannabinoid system tone is involved in the pathogenesis of obesity.^12^ Endocannabinoids exert their physiological functions in the body mainly via two receptors: cannabinoid receptors 1 and 2 (CB1 amd CB2).^13^ Some endocannabinoids-like compounds including oleoylethanolamide (OEA) are structurally similar to endocannabinoids but are incapable of binding to cannabinoid receptors.^14^

Oleoylethanolamide as an endogenous ethanolamide fatty acid is synthesized in the small intestine cells and adipose tissues,^15,16^ neurons^17^ and astrocytes^18^ and exerts its biological functions through other pathways instead of cannabinoid receptors.^19^ Oatmeal, nuts, and cocoa powder are the major foods sources of OEA in meals. However, the amount of OEA found in these foods is low (fewer than 2 µg/g).^20,21^ In addition to the protective effects of OEA in the many metabolic diseases including neurodegenerative disorders, anti-atherosclerosis effects, apoptosis, relieving pain, enhancement of lipid metabolism, cytoprotective actions on the pancreatic beta cells,^22-34^ decreasing inflammation and body weight are considered as other beneficial effects of OEA.^25-35^ In obese people, OEA can regulate the energy homeostasis and appetite mainly by activation of various receptors including proximal proliferator-activated receptor-α (PPAR-α), G-protein-coupled receptor 119 (GPR119) and transient receptor potential cation channel subfamily V (TRPV1). Indeed, OEA activates these receptors and delays meal initiation, reduces meal size, decreases intervals between meals and finally modulates body weight.^23,36-38^

According to experimental studies, OEA also suppresses the expression of IL-6, interleukin-8 (IL-8), intercellular adhesion molecule-1 (ICAM-1) and vascular cell adhesion molecule-1 (VCAM-1) in TNF-α induced inflammation in human umbilical vein endothelial cells through the activation of inflammatory receptors. OEA also inhibited the nuclear factor kappa-B (NF-kB) pathway in the body.^39^ In YT *et al's* survey, OEA (50 µmol/L) inhibited the TNF-α induced VCAM-1 expression in HUVEC.^40^

Considering the high prevalence of obesity and due to the lack of clinical studies assessing OEA´s potential in reducing inflammatory biomarkers and oxidative stress in obese people, the present study was aimed at investigating the effects of OEA supplementation on the inflammation and oxidative stress biomarkers in obese people.

## Materials and Methods

This randomized, double-blind, controlled clinical trial was done on 60 healthy obese people from November to May 2016 in Tabriz, Iran. Individuals between the ages of 18 and 59 years and with a body mass index (BMI) between 30 and 40 kg/m2 were defined as inclusion criteria. All participants were informed through the announcement and poster invitation and were recruited from clinics and healthcare centers of Tabriz University of Medical Science. Exclusion criteria were participants with current clinical problems including kidney diseases, liver and heart failure, gastrointestinal and rheumatic disorders, cigarette, smoking, pregnancy, and breastfeeding and menopause in women, participants taking antibiotics, probiotics and prebiotics supplements, weight loss drugs, omega 3 supplements, and multivitamin and mineral supplements during one month prior to the study.

The sample size was calculated according to the same variable (body mass) as a previous study.^41^ By considering the confidence interval 95%, power 90%, two-tailed test, and taking into account the 0.9% changes, the minimum sample size was calculated to be 26 healthy obese people in each group. Given that losses were possible in the follow-up period, 30 individuals were included in each group.

**Figure 1:**
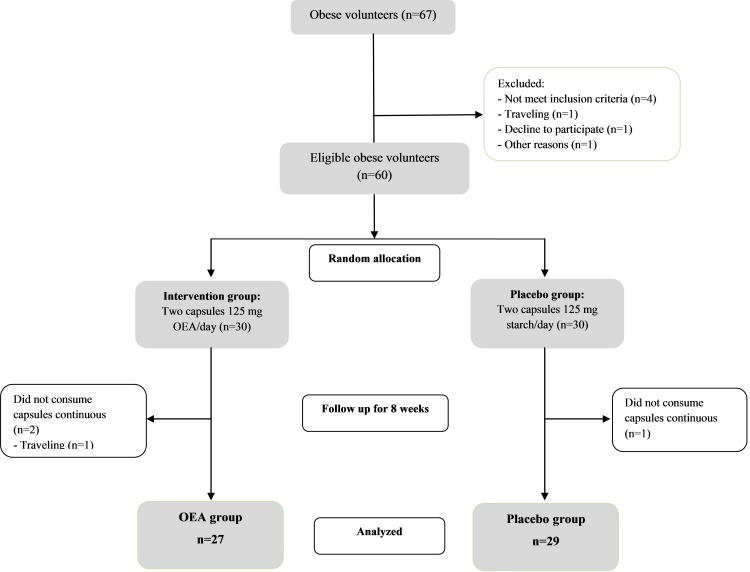
The flowchart of the study from baseline until the end of study

A written consent form completed by eligible participants at baseline. Demographic questionnaires including age, sex, occupational status, and educational level were completed by participants at baseline.All participants were randomly allocated to an intervention or a placebo group. The intervention group received two 125 mg OEA capsules daily 30-minutes before lunch and dinner meals (synthesized at the Nutrition Research Center, Tabriz University of Medical Science, Iran); the materials for synthesis were obtained from the Sigma-Aldrich Company (in detail was explained in our previous study)^42^ for 8 weeks. The placebo group received the same amount of placebo (starch). A third person who labeled capsules with identical shapes and colors with 2 codes remained unknown to the researchers until the end of the assays. Figure 1 shows how people were recruited and followed until the end of the study. All subjects were followed by weekly phone call to confirm that they consumed the capsules regularly. The data on those who consumed more than 90% of the capsules were analyzed.

### Anthropometric and biochemical assessments

At baseline, anthropometric measurements were taken in the fasting state. Body weights and heights were measured without shoes and with light clothing using a Seca scale (precision 100 g, Seca, Hamburg, Germany) and a stadiometer (Seca, Germany), respectively. The BMI was calculated by dividing weight/height2 (kg/m2).

Blood samples (5mL) were collected in vacuum collection tubes containing EDTA in the fasting state (10-12 hour, water permitted) at baseline and at the end of the study. The serums were isolated by high-speed centrifugation and were frozen immediately at −70 °C until assay. The concentration of malondialdehyde (MDA) and the total antioxidant status (TAS) were measured with a spectrophotometer using a commercial kit (Merck chemicals and Randox Laboratories, Ltd, Crumlin, UK, respectively). The Immunoturbidimetry assay and commercial kit were used to measure the high sensitive-C reactive protein level (Biosystems, Barcelona Spain COD 31927). The levels of IL-6 and TNF-α were also assayed using ELISA kits, according to the manufacturer’s instructions (IBL (REF BE53061 and REF BE55001, International GmbH, Hamburg, Germany).

### Statistical analysis

SPSS software was used to analysis of data (version 20; SPSS Inc., Chicago, IL). The normal distribution of variables was assessed by the Kolmogorov-Smirnov test. Numerical data was presented as mean (Standard deviation) and categorical data was presented as frequency (percentage). The baseline characteristics were compared by independent sample t tests (for quantitative variables) and the chi-squared test (for qualitative variables) in both groups. The within-group changes of IL-6, TNF-α, hs-CRP, MDA, and TA concentrations were assessed by paired sample t-test. We used analysis of covariance (ANCOVA) to detect difference between the intervention and placebo groups after adjusting for baseline measurements and confounder factors including age, sex, occupational and educational status. p< 0.05 was defined as statistically significant.

## Results and Discussion

Table 1 presents the baseline characteristics of participants in both groups. The analysis was performed on data from 56 participants who completed the intervention. Mean (SD) ages of subjects in the intervention group were 37.37 (8.74) and in the placebo group was 38.13 (9.28) years. About 55% and 65% of subjects in the intervention and placebo groups were females. No significant differences detected for all baseline variables with the exception of educational level (p>0.05). Participants reported no side effect or symptoms either during OEA treatment or at the end of the intervention.

**Figure 2:**
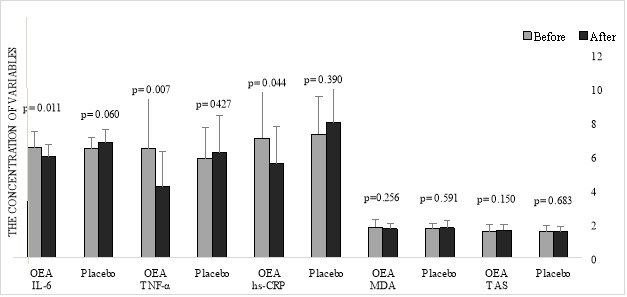
The Mean (SD) of inflammatory biomarkers and oxidative stress indices in the OEA and placebo groups (n=56)

Figure 2 shows the changes in the inflammatory biomarkers and oxidative stress indices before and after the intervention in both groups. The levels of IL-6 decreased significantly in the intervention group (before Mean (SD) = 6.51 (0.95), after Mean (SD) = 5.99 (0.72); p=0.011). Changes in the placebo group were not significant (p>0.05). According to the ANCOVA test, there was a significant difference between groups regarding the IL-6 variable, after adjusting for baseline value and demographic characteristics (p<0.001). TNF-α decreased significantly in the OEA group in comparison with baseline (before Mean (SD)=6.44 (3.19), after Mean (SD)=4.20 (2.19); p=0.007) and this result was confirmed by ANCOVA test (p=0.001).

The levels of hs-CRP in the OEA group decreased significantly at the end of the study (p=0.044) and a non-significant increase of this variable was detected in the placebo group (p>0.05). However, the ANCOVA test did not attain statistical significance. Changes in other biomarkers (MDA and TAS) were not significant (p>0.05). Table 2 presents the mean difference between inflammatory biomarkers and oxidative stress indices between groups after adjusting for baseline value and demographic characteristics.

Different dietary interventions have been identified to reduce inflammatory cytokines in chronic disorders.^43^ Indeed, dietary components act as ligands of the receptors for the activation of genes involved in the regulation of inflammatory responses.^44^ Oleoylethanolamide, a lipid mediator component, is a structural analog of the endocannabinoids with several beneficial effects in metabolic status.^41,45^

The present study investigated the potential anti-inflammatory effects of OEA in obesity. At the end of the study, the concentrations of IL-6 and TNF-α decreased significantly. However, the level of other biomarkers did not show significant changes between groups. Higher levels of IL-6 in obese people increases the expression of other acute phase proteins and systemic inflammation markers such as CRP.^46^ In agreement with this study, Anton *et al* investigated the anti-inflammatory effects of OEA in 87 rats exposed to alcohol. Supplementation of male Wistar rats (n=4-6 per group) with 5mg/kg OEA, resulted in a decrease of interleukin-1 β (IL-1 β), monocyte chemoattractant protein 1 (MCP-1), TNF-α levels and inhibition of pro-inflammatory enzymes including cyclooxygenase-2 (COX-2) and inducible nitric oxide syntheses (iNOS). Also, it has been reported that OEA had anti-depression effects and neuroprotective effects on alcohol intoxicated rats.^35^ In the study of Sun *et al*, the neuroprotective effects of OEA through the activation of PPAR-α receptors were reported. Supplementation of OEA (10mg/kg) increased the expression of I_k_B as NF_k_B-inhibitory protein in cortex cerebral mouse and inhibited the expression of COX-2 as NF_k_B-regulated protein.^26^ Zolese* et al* investigated the effects of OEA on lipid peroxidation and high-density lipoprotein (HDL)-associated paraoxonase activity (as an enzyme involved in antioxidant and anti-inflammatory pathways) in plasma samples derived from 13 healthy people. The results showed that incubation of plasma with 1µM OEA and 20-70µM CuSO_4_ and 10 mM (2,2 -Azobis-(2-amidinopropane) hydrochloride (AAPH) for 7 h protected plasma lipids and paraoxonase 1 enzyme activity against AAPH and copper-induced oxidation.^47^

**Table 1:** The demographic characteristics of obese people in the onset of study (n=56)

**Variables**	**OEA group (n=27)**	**Placebo group (n=29)**	**P** ^a^
Age (year)	37.37 (8.74)	38.13 (9.28)	0.572
Gender			
- Female	15 (55.6)	19 (65.5)	0.109
- Male	12 (44.4)	10 (34.5)	
Education level			
- Illiterate	13 (48.1)	15 (51.7)	0.010
-Diploma	3 (11.1)	11 (37.9)	
- Bachelor degree and above	11 (40.7)	3(10.3)	
Occupation			
- Clerk	8 (29.6)	4 (13.8)	0.438
- Housewife	14 (51.9)	19 (65.5)	
- Worker	5 (18.5)	6 (20.7)	
Weight (kg)	93.02 (13.22)	91.23 (13.61)	0.628
Height (cm)	163.73 (9.41)	160.96 (8.91)	0.340
BMI (kg/m^2^)	34.69 (2.41)	35.11 (2.82)	0.552
IL-6 (pm/mL)	6.51 (0.95)	6.43 (0.64)	0.698
TNF-α (pm/mL)	6.44 (3.19)	5.86 (1.85)	0.412
Hs-CRP (pm/mL)	7.04 (3.12)	7.28 (2.23)	0.666
TAS (mmol/L)	1.56 (0.37)	1.52 (0.37)	0.682
MDA (µmol/L)	1.79 (0.49)	1.72 (0.30)	0.533

^a^ Independent sample t-Test/ Chi2 test Data presented as Mean (SD)/ frequency (%)

In another study, the anti-inflammatory and neuroprotective properties of OEA were assessed in neuro-inflamed Wistar Hannover rats induced by lipopolysaccharide (LPS) (n=94, 5-6/group). OEA (10 mg/kg) prevented the significant up-regulation of NF_k_B and significantly reduced lipopolysaccharide-induced oxidative/nitrosative stress (p<0.001). Also, OEA disrupted lipopolysaccharide-induced anhedonia as the main symptom of a depressive-like state (p<0.001).^48^ In the study of Ma *et al*, the antioxidant and anti-inflammatory properties of OEA in 20 male mice were investigated. OEA (10 mg/kg/day) was administered orally for 8 weeks. The results showed that OEA prevented oxidative stress and endothelial cell damage, and inhibited early atherosclerotic plaque formation.^49^Table 3 summarized the results of studies regarding OEA supplementation on the inflammatory biomarkers and oxidative stress.

OEA exert its antioxidant and anti-inflammatory effects through different mechanisms. OEA acts as an endogenous ligand of the PPAR-α.^42,50^ PPAR-α is a member of the nuclear receptor proteins family and exerts effective roles in many metabolic pathways such as adipocytes differentiation and lipid metabolism.^51^ Regulating inflammatory responses was also attributed to the PPAR-α function. In macrophages, PPAR-α inhibits the scavenger receptor and nitric oxide synthesis.^39^ Also, PPAR-α reduces leukocyte adhesion to the activated endothelial cells and controls the migration of transendothelial leukocytes.^52^ Agonists of PPAR-α suppress the expression of IL-6 and COX-2 involved in the inflammatory pathways.^53^ OEA as a ligand of PPAR-α binds to the PPAR-α receptors and reduces the production of reactive oxygen species (ROS) and pro-inflammatory cytokines.^54^ OEA also inhibits the expression of VCAM-1 and ICAM-1 as molecules involved in the inflammatory responses.^55^ Other mechanisms involved in the cytoprotective and anti-inflammatory effects of OEA include the increasing activity of the anti-oxidative enzyme, reducing lipid peroxidation, and apoptosis-related proteins expression.^49^

To the best of the authors´ knowledge, this was the first clinical study to evaluate the unique effects of oleoylethanolamide on the inflammatory biomarkers and oxidative stress in obese people. The main limitations of this study include the insufficient purity of the synthesized OEA (~ 85%), not measuring OEA concentrations in the serum samples and the shorter duration of the supplementation.

**Table 2:** The effects of OEA supplementation on the inflammatory biomarkers and oxidative stress in obese people (n=56)

**Variables**	**OEA group (n=27)**	**Placebo group (n=29)**	**Mean Diff (95% CI)** **P** ^a^
IL-6 (pm/mL)			
Before	6.51 (0.95)	6.43 (0.64)	0.853 (0.447-1.258) <0.001
After	5.99 (0.72)	6.77 (0.78)	
TNF-α (pm/mL)			
Before	6.44 (3.19)	5.86 (1.85)	2.127 (0.935-3.319) 0.001
After	4.20 (2.09)	6.21 (2.20)	
hs-CRP (pm/mL)			
Before	7.04 (3.12)	7.28 (2.23)	1.590 (-0.242-3.422) 0.087
After	5.56 (2.16)	7.97 (2.41)	
MDA (µmol/L)			
Before	1.79 (0.49)	1.72 (0.30)	0.108 (-0.085-0.300) 0.267
After	1.70 (0.33)	1.76 (0.41)	
TAS (mmol/L)			
Before	1.56 (0.37)	1.52 (0.37)	0.093 (-0.210-0.025) 0.119
After	1.63 (0.32)	1.53 (0.34)	

Data were presented as Mean (SD) ^a^ Mean Diff (95% CI)P for ANCOVA test (Adjusting on Baseline values, age, gender, education, and occupation variables)

**Table 3:** The results of studies about the effects of oleoylethanolamide on the inflammatory biomarkers and oxidative stress

**Samples**	**Dose, Duration**	**Results**	**Reference**
Wild-type mice (C57BL/6)	OEA (10mg/kg) for 3 days	- The expression of IkB as NFkB-inhibitory protein in cortex cerebral of mouse increased - The expression of COX-2 as NFkB-regulated protein inhibited	Sun et al. (2007)
Healthy people (n=13)	OEA (1µM) CuSO4 (20-70µM) (2,2 -Azobis-(2-amidinopropane) hydrochloride (AAPH) (10 mM) for 7 hours	- Plasma lipids and paraoxonase 1 enzyme activity protected against AAPH and copper-induced oxidation by OEA	Zolese et al. (2008)
Vascular endothelial cell, healthy HUVEC and TNF-alpha induced HUVECs	OEA (10, 50 and 100 µmol.L) cultured at 37 degrees C for 7 h	- OEA (10 and 50 µmol.L) induced the CB2 protein and mRNA expression, but not 100 µmol.L. - TNF-alpha induced VCAM-1 expression and THP-1 adhere to HUVEC inhibited	YT et al, (2014)
Male mice (n=20)	OEA (10 mg/kg) administered orally 8 weeks	- Oxidative stress and endothelial cell damage prevented - Early atherosclerotic plaque formation inhibited	Ma et al. (2015)
Wistar Hannover rats (n=94, 5-6 /group) terated by LPS	OEA (10 mg/kg) 8 weeks	- Up-regulation of NFkB/ lipopolysaccharide-induced oxidative/nitrosative stress inhibited significantly (p<0.001) - Lipopolysaccharide-induced anhedonia as a core symptom of a depressive-like state disrupted (p<0.001)	Sayd et al, (2015)
Wistar rats exposed to alcohol (n=87, n=4-6 per groups)	OEA (5mg/kg)	- The levels of IL-1β, MCP-1, TNF-α decreased - Pro-inflammatory enzymes including COX-2 and iNOS inhibited - OEA had anti-depression effects and neuroprotective effects on alcoholic rats	Anton et al. (2017)

## Conclusion

In summary, daily supplementation of two 125 mg oleoylethanolamide in 56 healthy obese people for 8 weeks significantly decreased the levels of IL-6 and TNF-α; however, changes in MDA, TAS, and hs-CRP were insignificant. Considering the many beneficial effects of OEA, its use as a complementary agent could be effective in improving inflammation in obese people. However, future studies are needed to confirm the current results.

## Acknowledgments

The authors thank the Department of Nutrition, Faculty of Health and Nutrition. This is a part of a database from Ph.D. thesis entitled "The effect of oleoylethanolamide supplementation on PPAR-α gene expression, some inflammatory biomarkers and the abundance of Akkermansia muciniphila bacteria in the stool of obese people: A double-blind randomized placebo-controlled clinical trial".

## Ethical Issues

A written consent form completed by eligible subjects. The regional ethics committee of the Tabriz University of Medical Science approved the whole protocol of the study and allocated the number code IR.TB MED.REC.1395.618. The study was registered in the Iranian Registry of Clinical trial center with number IRCT201607132017N30 with URL: www.IRCT.IR.

## Conflict of Interest

The authors declare there is no conflict of interest in the content of this study.
